# Embryo migration following ART documented by 2D/3D ultrasound

**Published:** 2020-08-05

**Authors:** SH Saravelos, DT Balfoussia, GWS Kong, JPW Chung, JSM Mak, CHS Chung, LP Cheung, TC Li

**Affiliations:** Assisted Reproductive Technology Unit, Prince of Wales Hospital, The Chinese University of Hong Kong, Hong Kong;; Wolfson Fertility Centre, Hammersmith Hospital, Imperial College Healthcare NHS Trust, W12 0HS, London, UK;; Department of Obstetrics and Gynaecology, Hillingdon Hospital, UB8 3NN, London, UK.

**Keywords:** Embryo migration, ART, ectopic pregnancy, ultrasound

## Abstract

Embryos have traditionally been thought to implant at the exact site they are transferred during assisted reproductive technology (ART). The introduction of 2D/3D ultrasound has allowed for mapping of the transfer site using air bubbles as a surrogate marker of embryo location. This study’s aim was to compare the location of embryo transfer (ET) on ultrasound to that of embryo implantation. We present four cases of ectopic pregnancy at four sites: tubal, cervical, interstitial and ovarian. We compare the site of implantation on 2D/3D ultrasound at six weeks of pregnancy to that of transfer as assessed on 2D/3D ultrasound. In all four cases, the embryo flash was visualised in the centre of the uterine cavity on ultrasound at ET. At six weeks of pregnancy, the uterine cavity was empty and an ectopic pregnancy was identified. The tubal and ovarian ectopics were managed surgically whilst the cervical and interstitial pregnancies were treated with systemic methotrexate. These cases demonstrate embryo implantation distal to the ultrasound-confirmed site of transfer. These cases provide visually compelling evidence of embryo migration following ET and lend support to the theory that ectopic pregnancy may occur as a result of embryo migration, rather than poor ET technique.

## Introduction

### 

For some time now it has been thought that the majority of embryos implant at the location where they are transferred in women undergoing assisted reproductive technology (ART) treatment. This was first supported by the novel work of Baba et al. (2000), who showed that approximately 80% of embryos appeared to implant in the areas where they had been originally transferred following IVF-ET cycles . This assumption had not really been challenged for over 15 years, and in fact a plethora of studies were subsequently published reinforcing this notion by reporting a correlation between the site of ET and the pregnancy rates (Lambers et al., 2007; Friedman et al., 2011; Frankfurter et al., 2004; Pacchiarotti et al., 2007; Coroleu et al., 2002; Oliveira et al., 2004; Tiras at al., 2010; Franco et al., 2004; Cenksoy et al., 2014). Further novel work using 3D ultrasound (US) was also published around that time, which highlighted the possibility of precisely mapping the site of ET by identifying both the catheter and air bubbles (the so called ‘embryo flash’) on the reconstructed coronal plane of the uterus (Gergely et al., 2005). This was followed on by reports suggesting that clinical outcomes may be improved by this technique, through achieving a more precise placement of the embryo under 3D US guidance (Fang et al., 2009). With these findings in mind, our team recently investigated the importance of both the location of the embryo flash on 3D US, and also its movement within the uterine cavity for over an hour following ET. Interestingly, it was found that the location of the embryo flash and the direction of its movement at 60 minutes, but not at 1 or 5 minutes after transfer were associated with clinical pregnancy (Saravelos et al., 2016a). Subsequently, using 3D US to assess the patients who achieved a clinical pregnancy, we found that only about half of the embryos implanted at the exact location where they had been initially transferred (Saravelos et al., 2016b). Despite the obvious limitation of using the air bubbles as a surrogate marker for the actual location of the embryo, these findings did challenge the traditional notion that the embryo will generally implant at the exact position where it has been transferred. More importantly, it somewhat highlighted the concept of embryo migration post ET.

### Case 1

The first case concerns a 38-year-old woman with primary infertility of six years due to tubal factor. She underwent a laparoscopic peritoneal adhesiolysis with bilateral salpingostomy. She subsequently underwent a first cycle of IVF two years later with 300 IU daily of human menopausal gonadotrophin (hMG) (Pergonal, Merck Serono, Germany) stimulation and antagonist downregulation (Cetrotide, Merck Serono, Germany). Four oocytes were retrieved 36 hours following trigger with 10,000 IU of hCG, of which three were successfully fertilised via conventional IVF. All three reached the cleavage stage, and two were selected for fresh cycle transfer (8 cell and 9 cell with <10% fragmentation), while the remaining one was cryopreserved. Luteal phase support was provided in the form of vaginal progesterone (Endometrin 100 mg twice daily, Ferring, Switzerland) from the day of oocyte retrieval.

There was no sonographic evidence of hydrosalpinx on the day of embryo transfer. With regards to the ET, following a moderately full bladder, the patient was placed in a lithotomy position, where a Cusco’s speculum was inserted into the vagina and the cervical mucus was cleared using a sterile cotton wool stick. Embryos were then loaded into an atraumatic Cooks Guardia Access EchoTip catheter (Cook Medical, IN, USA) by using 20 μl of culture medium containing the embryos, followed by a drop of air, followed by a further drop of culture medium. The ET catheter was then inserted through the cervix under US guidance (Voluson 730 Expert, GE Medical Systems Kretztechnik GmbH & Co, Austria), aiming to place the inner echogenic catheter tip at the centre of the uterine cavity. When that had been achieved, the embryos were injected slowly under direct US vision. Assessment by 3D US was performed to precisely map the location of the embryo flash (i.e. air bubbles) within the uterine cavity. The inner followed by the outer catheter were then removed under US guidance and returned to the embryologist to confirm that the transfer had been carried out successfully.

**Figure 1:**
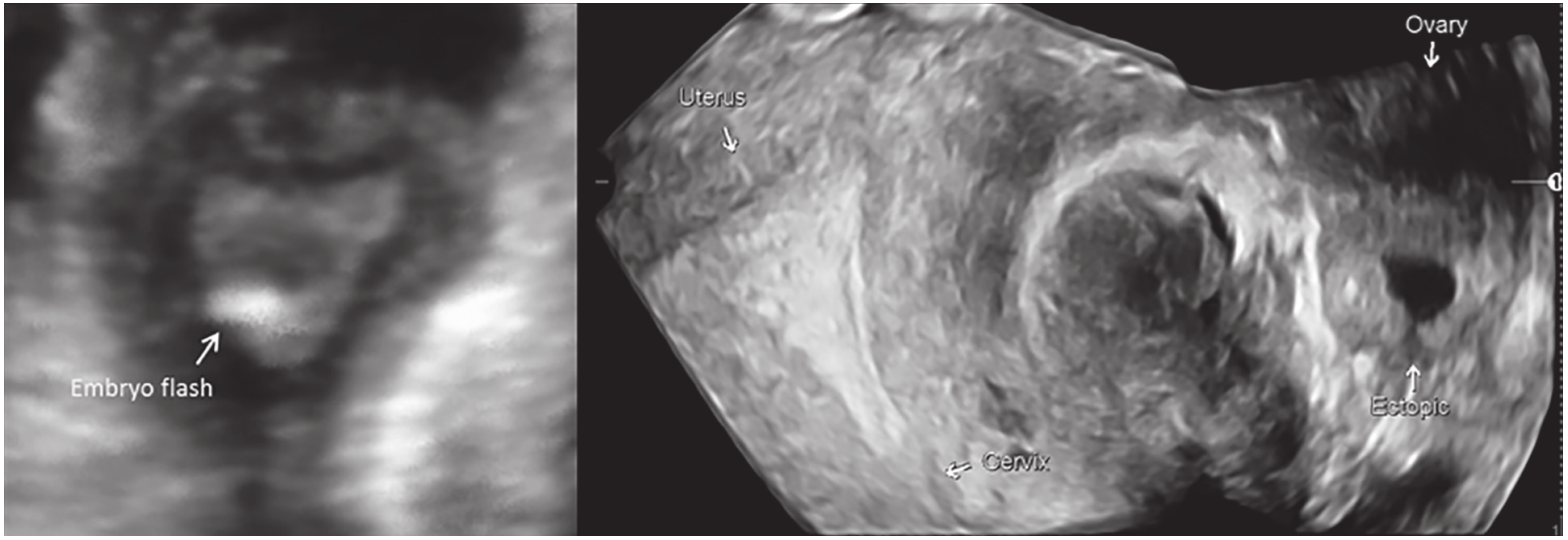
— The initial site of embryo transfer followed by the eventual site of embryo implantation (day 28) as documented with 3D US. There is a significant retrograde migration of the embryo from the lower right region of the uterine cavity to the distal region of the left fallopian tube.

Serum hCG measured on day 14 following fertilisation confirmed a positive pregnancy (37 IU/L), with a suboptimal rise on day 21 (144 IU/L). US examination on day 28 revealed a left tubal ectopic pregnancy of 14x17 mm, while the hCG level had reached 3239 IU/L. 3D US examination (Voluson E8 Expert, GE Medical Systems, Zipf, Austria) using OmniView rendering (Polyline function with 1mm VCI slice) confirmed an empty uterine cavity with the left tubal ectopic pregnancy appearing adjacent to the left ovary several centimetres away from the initial site of ET. Following counselling, the patient underwent a laparoscopy, which confirmed an ectopic pregnancy in the distal ampullary region of the left fallopian tube. The initial site of ET along with the eventual site of implantation are shown clearly in Figure 1.

### Case 2

The second case concerns a 39-year-old woman with secondary infertility due to tubal factor and a compromised ovarian reserve. She had undergone a previous termination of pregnancy almost 20 years ago, but had failed to conceive in the last 5 years despite unprotected intercourse. HSG revealed bilateral hydrosalpinges, which were both removed in a subsequent laparoscopy. Her first IVF cycle in 2015 was cancelled due to a poor response to ovarian stimulation. She subsequently underwent a second IVF cycle in 2016 with 300 IU daily of recombinant follicle stimulating hormone (rFSH) (Gonal-F, Merck Serono, Germany) stimulation and antagonist downregulation (Cetrotide, Merck Serono, Germany). Three oocytes were retrieved 36 hours following hCG trigger, of which two were fertilised via conventional IVF, with one reaching the cleavage stage (7 cell with <10% fragmentation). Luteal phase support was provided in the form of vaginal progesterone (Endometrin 100 mg twice daily, Ferring, Switzerland) from the day of oocyte retrieval.

Single ET was performed on day 3 of fertilisation in the same manner and technique as described above. The inner echogenic catheter tip was placed at the centre of the uterine cavity, followed by the controlled injection of the embryo under direct US vision. Assessment by 3D US was again performed to precisely map the location of the embryo flash (i.e. air bubbles) within the uterine cavity. The inner followed by the outer catheter were then removed under US guidance and returned to the embryologist who confirmed the absence of any retained embryos. In this case, there was no US evidence of air bubbles or medium in the lower uterine cavity or cervix following the removal of both catheters.

**Figure 2:**
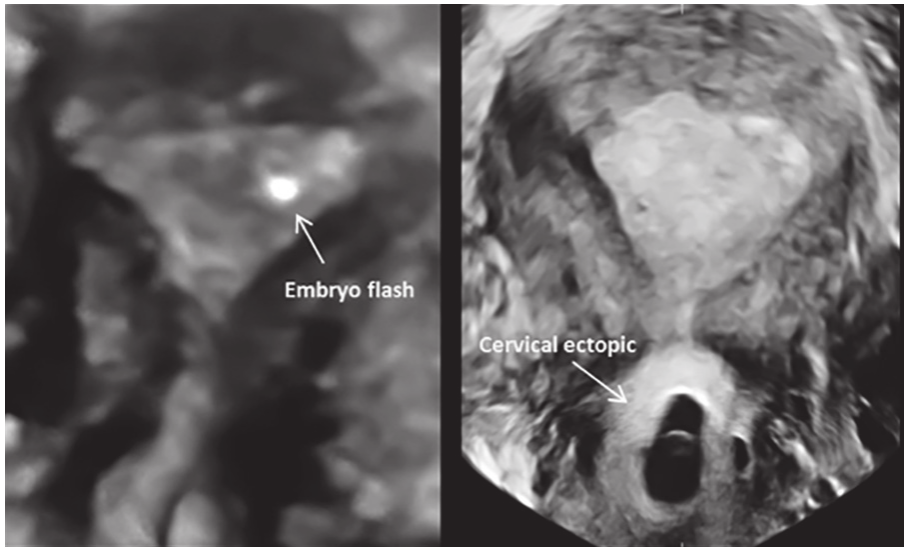
— The initial site of embryo transfer followed by the eventual site of embryo implantation as documented with 3D US. There is a significant antegrade migration of the embryo from the upper left region of the uterine cavity to the mid- region of the endocervical canal.

Following the ET, serum hCG measured on days 14 and 21 of fertilisation, confirmed a growing gestation (levels 103 IU/L and 1489 IU/L respectively). Unfortunately, US examination on day 28 revealed a live cervical ectopic pregnancy with gestational sac, yolk sac and crown-rump measurements of 9.6 mm, 2.3 mm and 1.4 mm respectively. Subsequent 3D US examination using OmniView rendering (Polyline function with 1 mm VCI slice) confirmed an empty uterine cavity with the an ectopic pregnancy with fetal cardiac pulsations appearing in the mid-part of the cervical canal, several centimetres away from the initial site of ET, and in fact lower than even the placement of the outer ET catheter. The internal os appeared closed on ultrasound. Following counselling, the patient proceeded with systemic methotrexate injection, which resulted in an uneventful resolution of the pregnancy. The initial site of ET along with the eventual site of implantation is shown clearly in Figure 2.

### Case 3

The third case concerns a 32-year-old woman with four years of primary infertility due to male factor. She underwent her first ART cycle in 2015 with 375 IU daily of recombinant follicle stimulating hormone (rFSH) (Gonal-F, Merck Serono, Germany) stimulation and GnRH antagonist downregulation (Cetrotide, Merck Serono, Germany). Eight oocytes were retrieved 36 hours following hCG trigger and were fertilised via ICSI. Following culture for 5 days, a single early blastocyst was selected for transfer, while two surplus embryos were cryopreserved. Luteal phase support was provided in the form of vaginal progesterone (Endometrin 100 mg twice daily, Ferring, Switzerland) from the day of oocyte retrieval as in the previous cases.

ET was performed in the same manner and technique as described above. The inner echogenic catheter tip was placed at the centre of the uterine cavity, followed by the controlled injection of the embryo under direct US vision.

The inner followed by the outer catheter were then removed under US guidance and returned to the embryologist who carefully examined them for any retained embryos. Assessment by 3D US this time revealed the embryo flash (i.e. air bubbles) to be clearly within the lower uterine cavity, while it was evident that the inner catheter had never reached the region of either cornua.

**Figure 3:**
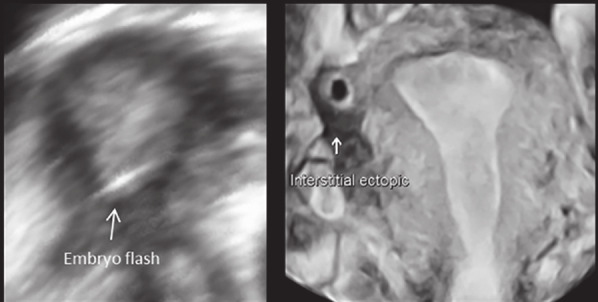
— The initial site of embryo transfer followed by the eventual site of embryo implantation as documented with 3D US. There is a significant retrograde migration of the embryo from the lower region of the uterine cavity to the interstitial region of the right fallopian tube.

Following the ET, serum hCG measured on day 14 of fertilisation confirmed a positive pregnancy (level of 617 IU/L). Unfortunately, US examination on day 28 was suspicious of an ectopic pregnancy with a gestational sac of 3.6 mm. Subsequent 3D US examination (OmniView function with 1 mm VCI slice) confirmed an empty uterine cavity with a right interstitial ectopic pregnancy that had implanted significantly higher than the initial site of ET. Following counselling, the patient underwent successful medical management with systemic methotrexate, with the hCG level became undetectable within the next 8 weeks. The initial site of ET along with the eventual site of implantation is shown clearly in Figure 3.

### Case 4

The fourth case concerns a 29-year old woman who presented with a five-year history of primary subfertility secondary to endometriosis and tubal factor. Her hysterosalpingography (HSG) demonstrated bilateral tubal patency but with a dilated left fallopian tube, which was not visible on US. The patient declined pre-treatment laparoscopy. She underwent a fresh IVF cycle, followed by a frozen ET cycle, which ended in a biochemical pregnancy loss. She subsequently underwent a vitrified blastocyst transfer cycle with oestrogen replacement patches (Evorel ® 100 mcg/day on alternate days, increased to 200 mcg/ day after 7 days, Janssen-Cilag Ltd) and GnRH antagonist (Ganirelix ® 0.25 mg, Orgalutran, N.V. Organon, Netherlands) for the first seven days.

The endometrium measured 14 mm, thirteen days post commencement of oestrogen. Progesterone supplementation was commenced subucutaneously (Lubion ® 25 mg once daily, IBSA, Italy) and embryo transfer of a 5BB blastocyst took place six days later. There was no hydrosalpinx visible on ultrasound on the day of transfer. The embryo had re-expanded post thaw and there was over 95% survival of vitrified cells. The transfer procedure was performed under US guidance as previously described, using an atraumatic Wallace ® Classic transfer catheter (Cooper Surgical, Denmark). The inner catheter dip was advanced until mid-uterine cavity and the embryo was injected under direct US vision. The inner followed by the outer catheter were then removed and returned to the embryologist to confirm that the embryos were not retained. Assessment by US revealed the embryo flash to be visible in the mid-cavity of the uterus.

**Figure 4:**
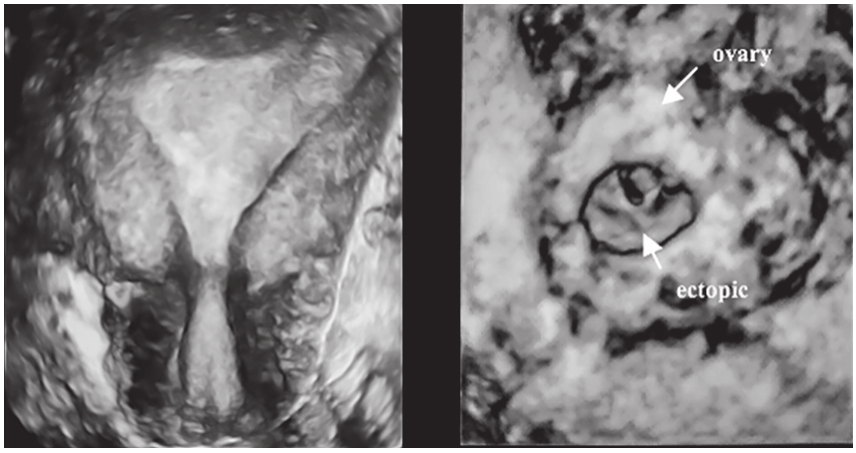
— T3D US at 6 weeks gestation following an uncomplicated embryo transfer demonstrating an empty uterine cavity and an ovarian ectopic implantation with a clear formation of a gestational sac, yolk sac and embryonic pole.

Urine pregnancy test twelve days later was positive, however, the patient complained of bleeding at five weeks of gestation. Serum hCG at 5+3 weeks was 5,004 IU/L and 8,482 IU/L 48 hours later. US at six weeks of pregnancy identified a live left ovarian ectopic pregnancy with a gestation sac, yolk sac and fetal pole measuring 7.8 mm, 4 mm and 1.9 mm respectively, whilst the serum hCG had reached 13,727 IU/L. 3D US examination (Voluson E10, GE Medical Systems, Zipf, Austria) using Omniview rendering confirmed an empty uterine cavity and a left ovarian ectopic. Following counselling, the patient underwent emergency laparoscopy which confirmed the US findings. There was severe endometriosis, a small amount of haemoperitoneum and the left ovary was seen to contain an ectopic pregnancy and was adherent to both a clubbed left fallopian tube and to the sigmoid colon. Conversion to laparotomy was undertaken in view of the significant adhesions with the patient undergoing a left salpingo-oophorectomy. The image from the site of pregnancy implantation is shown in Figure 4.

## Discussion

Recent literature is beginning to question the traditional notion that the majority of embryos will implant at the location where they have been transferred. The most intuitive explanation would appear to be embryo migration. Unfortunately, it is difficult to gauge the precise incidence and degree of embryo migration, as the embryo itself cannot be directly visualised following transfer; this leaves the air bubbles adjacent to the embryo as the best surrogate marker available to examine. In this article, we present four cases, whereby 2D/3D US mapping of both air bubbles and subsequent implanted gestations provide visually compelling evidence of embryo migration following ET.

Interestingly, the first reference to the notion of embryo migration was made by Steptoe and Edwards who in 1976 reported a tubal pregnancy that occurred because the embryo “was carried into the oviduct rather than the uterine cavity”, despite them having performed an intrauterine ET (Steptoe and Edwards, 1976). Although subsequent studies have gone on to examine the migration of contrast fluid within the uterine cavity following mock ET (Zhu et al., 2014), to our knowledge there has been only one report to have provided visual documentation of significant embryo migration following actual ET. In this intriguing article by Soares et al, 2D US imaging was used to demonstrate how an embryo that had been transferred to the left horn of a bicornuate uterus eventually implanted in the right horn of that uterus (Soares et al., 2008). This led the authors to question whether the initial location of the ET in fact indicates where the embryo will later implant. To our knowledge following the publication by Soares et al there have been no other reports describing significant embryo migration following ET. Furthermore, there appear to be no reports containing a visual depiction of embryo migration resulting in a tubal, cervical or ovarian ectopic implantation. The rarity of documenting such cases, with 3D US in particular, can be attributed to the approximately 1% and 0.01% estimated incidence for tubal and cervical ectopic pregnancies respectively (Santos- Ribeiro et al., 2016; Verma et al., 2009). This would require a unit with a 50% pregnancy rate to perform approximately 200 and 20,000 ETs under 3D US guidance, to capture one tubal and one cervical ectopic pregnancy respectively.

Our data appear compelling but certain considerations need to be borne in mind. Firstly, demonstrating embryo migration using air bubbles has the caveat of reliance on these as surrogate markers of embryo location, which may not always be the case. This is particularly significant for the case of the cervical ectopic pregnancy. Secondly, one of the cases presented involved transfer of a cleavage stage embryo, potentially posing a higher risk of embryo migration compared to blastocyst transfer, as implantation only takes place after the embryo has hatched. Furthermore, in the presented cases series, there were no ultrasound-visible hydrosalpinges at the time of ET. This is significant as there is evidence that a hydrosalpinx can result in abnormal uterotubal movements causing embryo migration into the fallopian tube and subsequent tubal ectopic pregnancy (Garde et al., 2006). Our data should therefore be interpreted with caution as embryo migration may not occur similarly, or have the same impact, in women with versus without tubal factor infertility. Furthermore, uterine contraction pattern during embryo transfer was not measured in this case series. This is potentially significant as increased uterine contractions during embryo transfer have been linked to lower pregnancy rates (Chung et al., 2016). If these were demonstrated to contribute to embryo migration and result in ectopic pregnancy, this could constitute an additional argument for consideration of a muscle relaxant in a select subgroup of patients at the time of embryo transfer.

The demonstration of embryo migration has important implications for clinical practice as it may reassure clinicians and patients that ectopic pregnancies do not necessarily occur due to poor ET technique. When looking at these four carefully documented cases of different types of ectopic pregnancy, the US findings would suggest that the most likely culprit for ectopic implantation is in fact embryo migration rather than poor ET technique. In addition to the implications for clinical practice, this concept of embryo migration may bear equally important implications for future research in ART. Despite recent advancements in the screening of embryos, implantation failure still continues to occur even with high quality and/or euploid embryos (Coughlan et al., 2014). Although this may be due to a number of different potential pathophysiological factors, embryo migration may also need to be considered as a contributory mechanism. Given that we can observe embryo migration in women who achieve implantation, it is plausible that where implantation is unsuccessful this may be due to embryos that have undergone such significant migration to the extent of expulsion outside the uterine cavity.

In conclusion, we present four carefully documented cases of different types of ectopic pregnancy following ART that provide compelling visual evidence of significant embryo migration following ET. Although it is difficult to ascertain the extent at which embryo migration occurs, it is possible that a significant proportion of ectopic pregnancies occur as a result of embryo migration rather than poor ET technique.
